# Targeted metabolomics profiles are strongly correlated with nutritional patterns in women

**DOI:** 10.1007/s11306-012-0469-6

**Published:** 2012-10-06

**Authors:** Cristina Menni, Guangju Zhai, Alexander MacGregor, Cornelia Prehn, Werner Römisch-Margl, Karsten Suhre, Jerzy Adamski, Aedin Cassidy, Thomas Illig, Tim D. Spector, Ana M. Valdes

**Affiliations:** 1 Department of Twin Research & Genetic Epidemiology, King’s College London, St Thomas Hospital, London, SE17EH UK; 2 Faculty of Medicine, Memorial University of Newfoundland, St John’s, NL Canada; 3 Norwich Medical School, University of East Anglia, Norwich, UK; 4 Helmholtz Zentrum München, Institute of Experimental Genetics, Genome Analysis Center, Neuherberg, Germany; 5 Helmholtz Zentrum München, Institute of Bioinformatics and Systems Biology, Neuherberg, Germany; 6 Department of Physiology and Biophysics, Weill Cornell Medical College in Qatar, Education City, Qatar Foundation, Doha, State of Qatar; 7 Faculty of Biology, Ludwig-Maximilians-Universität, Großhaderner Str. 2, Planegg-Martinsried, Germany; 8 Lehrstuhl für Experimentelle Genetik, Technische Universität München, Freising-Weihenstephan, Germany; 9 Research Unit of Molecular Epidemiology, Helmholtz Zentrum München, Neuherberg, Germany; 10 Hannover Unified Biobank, Hannover Medical School, Hannover, Germany

**Keywords:** Metabolomics, Twins, Dietary pattern, Nutrition habits, Food questionnaires

## Abstract

Nutrition plays an important role in human metabolism and health. Metabolomics is a promising tool for clinical, genetic and nutritional studies. A key question is to what extent metabolomic profiles reflect nutritional patterns in an epidemiological setting. We assessed the relationship between metabolomic profiles and nutritional intake in women from a large cross-sectional community study. Food frequency questionnaires (FFQs) were applied to 1,003 women from the TwinsUK cohort with targeted metabolomic analyses of serum samples using the Biocrates Absolute-IDQ™ Kit p150 (163 metabolites). We analyzed seven nutritional parameters: coffee intake, garlic intake and nutritional scores derived from the FFQs summarizing fruit and vegetable intake, alcohol intake, meat intake, hypo-caloric dieting and a “traditional English” diet. We studied the correlation between metabolite levels and dietary intake patterns in the larger population and identified for each trait between 14 and 20 independent monozygotic twins pairs discordant for nutritional intake and replicated results in this set. Results from both analyses were then meta-analyzed. For the metabolites associated with nutritional patterns, we calculated heritability using structural equation modelling. 42 metabolite nutrient intake associations were statistically significant in the discovery samples (Bonferroni *P* < 4 × 10^−5^) and 11 metabolite nutrient intake associations remained significant after validation. We found the strongest associations for fruit and vegetables intake and a glycerophospholipid (Phosphatidylcholine diacyl C38:6, *P* = 1.39 × 10^−9^) and a sphingolipid (Sphingomyeline C26:1, *P* = 6.95 × 10^−13^). We also found significant associations for coffee (confirming a previous association with C10 reported in an independent study), garlic intake and hypo-caloric dieting. Using the twin study design we find that two thirds the metabolites associated with nutritional patterns have a significant genetic contribution, and the remaining third are solely environmentally determined. Our data confirm the value of metabolomic studies for nutritional epidemiologic research.

**Electronic supplementary material:**

The online version of this article (doi:10.1007/s11306-012-0469-6) contains supplementary material, which is available to authorized users.

## Introduction

Nutrition plays an important role in human metabolism and health. Metabolomics is considered a promising tool for nutritional studies (Oresic 2009). It aims to profile all low-molecular weight metabolites that are present in biological samples to enhance the understanding of the effect of a particular stimulus on metabolic pathways (Brennan 2008).

Combining the data obtained with multivariate data analysis tools allows the exploration of changes induced by a biological treatment or changes resulting from a particular phenotype. The interest in using metabolomics for nutritional research has increased recently given the intimate relationship between nutrients and metabolism (Brennan 2008). A key question that needs to be answered is to what extent do metabolomic profiles reflect nutritional patterns.

Some interventional studies have examined the role of specific foods or nutrients on metabolite patterns. For example, Llorach et al. (2010) assessed the role of a single dose of almond skin extract on the urine metabolome of 24 volunteers (12 who ingested a dietary supplement and 12 who ingested a placebo) over a 24 h period. The study identified 34 metabolites associated with the single dose of almond extract. Similar study designs have been applied to investigate the role of meat intake (Cross et al. 2011; Stella et al. 2006) and the role of three distinct dietary patterns (O’Sullivan et al. 2011) on a limited number of urinary biomarkers. Some observational studies have also been conducted. Altaimer et al. ran two observational studies in 284 male individuals to investigate the role of coffee consumption and different nutrients on serum metabolites. Coffee consumption was found to significantly correlate with metabolomic profilings (Altmaier et al. 2009) and dietary pattern highly associated with serum metabolite concentrations were identified (Altmaier et al. 2011) thus showing that metabolites are reliable candidates for risk assessment in prospective epidemiologic studies with blood sample collection at one time point and that it is highly relevant to explore the link between nutrient intake and metabolite patterns in an epidemiological (non interventional) setting.

The AbsoluteIDZ kit developed by Biocrates Life Science AG (Innsbruk, Austria) allows more than 160 targeted metabolites to be quantified in over four compound classes. The kit measures the targeted metabolites in an easy, reliable and robust way. Also, the 4 months reproducibility is good and a single measurement appears to be sufficient for risk assessment in epidemiologic studies with healthy subjects Floegel et al. (2011). In a previous study on 2,362 female twins, five distinct dietary patterns that best represent the sources of independent variation in dietary intake in the twins, were identified using food frequency questionnaires (FFQs): fruit and vegetables, high alcohol consumption, traditional English diet, dieting and low meat (Teucher et al. 2007). FFQ are particularly strong at detecting patterns consistently, and principal component analysis (PCA) derived pattern cores are widely used to assess association with health outcome, for example cardiovascular disease, stroke and the risk of cancer (Stricker et al. 2012; Schulze et al. 2001; Adebamowo et al. 2005; Ouderaa et al. 2006).

In the present study we assessed the role of dietary intake patterns on metabolomic parameters, also including foods (e.g. garlic) that are commonly perceived as related to an individual’s health in the context of complex chronic diseases such as metabolic syndrome or cardiovascular disease. Using a targeted metabolomics approach we analysed the correlation between metabolite serum levels using the Biocrates Absolute-IDQ™ Kit p150 and the above five dietary patterns in 892–965 individuals. These patterns essentially cover all relevant dietary intake information available in the FFQs and are broadly comparable to those reported in a number of large-scale population-based studies of nutrition (Stricker et al. 2012; Schulze et al. 2001; Adebamowo et al. 2005; Ouderaa et al. 2006). We also tested for coffee intake to be able to compare our data to previously published studies (Altmaier et al. 2009) and for garlic intake since, it has been highlighted as a beneficial dietary constituent (Baños et al. 2008). A recent study (Suhre et al. 2011) has shown the strong statistical power available even with relatively modest sample sizes to identify genes underlying metabolite circulating levels. However, not all metabolites are necessarily influenced by an individual’s genetic make-up. Having identified metabolites associated with dietary intake patterns or dietary components, we validated the results using identical twins discordant for that particular nutritional intake or dietary component. Moreover, we evaluated the heritability of these metabolites. The rationale of this study therefore is to provide investigators with some key information necessary to pursue the study of the molecular mechanisms that underlie the relationship between dietary patterns and health outcomes, including information on the genetic contribution to the metabolites that relate to dietary intake patterns.

## Subjects and methods

### Study population

Study subjects were twins enrolled in the TwinsUK registry, a national register of adult twins. Twins were recruited as volunteers by successive media campaigns without selecting for particular diseases or traits (Moayyeri et al. 2012). In this study we analysed data from 1,003 female twins who completed the FFQs and had metabolomic data available. The study was approved by St. Thomas’ Hospital Research Ethics Committee, and all twins provided informed written consent.

### Dietary and other data

Twins were sent, by post, the 131-item FFQ which was developed for the EPIC (European Prospective Investigation into Cancer and Nutrition) Norfolk study (Bingham et al. 2001). Macro and micro nutrient intakes were calculated from an established nutrient database (Holland et al. 1991). For each food group, the frequency of intake (serving/wk) was adjusted for the total energy intake using the residual method. The energy-adjusted intakes were standardised and used in the PCA as previously described (Teucher et al. 2007). Dietary patterns were captured by five principal components of food consumption which accounted for 22 % of the total variance: fruit and vegetable, high alcohol, traditional English diet, hypo-caloric dieting and low meat. The five dietary patterns are PCA-generated scores. As such, they are independent variables standardized to have a mean of zero and a SD of one in the whole TwinsUK study population. Each dietary pattern should be considered as the representative of a particular food pattern intake. For instance, a positive fruit and vegetable score can be understood as an average high consumption of fruit, allium and cruciferous vegetable together with a low consumption of fried potatoes. Vice versa, a negative score may be taken to mean low consumption of fruit, allium and cruciferous vegetables together with high consumption of fried potatoes. Garlic and coffee are measured in terms of estimated weekly consumptions. Body mass index (BMI) was calculated as body weight in kilograms divided by the square of height in square meters.

### Metabolomic measurements

The serum samples were collected after an overnight fast of all the study subjects. They were stored in −80º C freezers from which they were retrieved and sent to Germany for metabolite measurements. A targeted metabolomic assay was done in samples of fasting serum from participants in the British TwinsUK study (*n* = 1,003) using the Biocrates Absolute IDQ™-kit p150 (BIOCRATES Life Sciences AG, Innsbruck, Austria), as previously described (Illig et al. 2010; Mittelstrass et al. 2011; Römish-Margl et al. 2012). Briefly, the flow injection analysis (FIA) tandem mass spectrometry (MS/MS) method is used to quantify 163 known small molecule metabolites simultaneously by multiple reaction monitoring. Quantification of the metabolites is achieved by reference to appropriate internal standards. Reproducibility of the assay was performed in 23 serum samples. The mean of the coefficient of variation (CV) for the 163 metabolites was 0.07 and 90 % of the metabolites had a CV of <0.10.

Concentrations of all analysed metabolites are reported in μM.

### Metabolites panel

The metabolomics dataset contains 41 acylcarnitines (C*x*:*y*), hydroxylacylcarnitines [C(OH)*x*:*y*] and dicarboxylacylcarnitines (C*x*:*y*-DC); 14 amino acids; one Sugar; 15 sphingomyelins (SM*x*:*y*) and sphingomyelin-derivatives [SM(OH)*x*:*y*]; and 92 glycerophospholipids (PC). Glycerophospholipids are differentiated with respect to the presence of ester (a) and ether (e) bonds in the glycerol moiety, where two letters (aa = diacyl, ae = acyl-alkyl) denote that two glycerol positions are bound to a fatty acid residue, while a single letter (a = acyl) indicates the presence of a single fatty acid residue. Lipid side chain composition is abbreviated as C*x*:*y*, where *x* denotes the number of carbons in the side chain and *y* the number of double bonds. The full list of metabolites is presented in the supplementary material (Table S1).

### Statistical analysis

Statistical analysis was carried out using Stata version 11. The metabolite serum concentrations were first log transformed as these were not normally distributed, but right-skewed. For each dietary variable, linear regression analysis (adjusting for age and BMI) was first undertaken by excluding MZ discordant twins (i.e. MZ twins with measures one SD apart).

Familial relatedness was accounted for using random intercept linear regression:

1 $$ Y_{i} = \beta_{0} + \beta_{i} X_{ij} + \gamma_{i} age_{ij} + \delta_{i} BMI_{ij} + \zeta_{j} + \varepsilon_{ij} $$

where Y_*i*_ and X_*ij*_ are respectively the log-transformed metabolite (Y) and the dietary variable of twin *j* from pair *i*. ζ_*j*_ is the family-specific error component which represents the omitted family characteristics or unobserved heterogeneity. So the comparison between metabolite and dietary variables was performed between each twin pair.

We then ran for each significant metabolite-dietary variable (3 × 10^−4^ = 0.05/162) the same linear regression analysis on the discordant MZ twin pairs. Finally, we combined results using an inverse variance fixed effect meta-analysis. The fixed effect model provides a weighted average of the study estimates, the weights being the inverse of the variance of the study estimate. We used Bonferroni correction to account for multiple testing thus giving a significant threshold of 4 × 10^−5^ (0.05/7 nutrients × 163 metabolites).

For the metabolites associated with nutritional patterns, we first calculated the intra-class correlation coefficient (ICC) for MZ and DZ pairs. We then estimated heritability using structural equation modelling to separate the observed phenotypic variance into three latent sources of variation: additive genetic variance (A), shared/common environmental variance (C), and non-shared/unique environmental variance (E) (Neale and Cardon 1992). Additive genetic influences are indicated when MZ twins are more similar than DZ twins. The common environmental component estimates the contribution of family environment, which is assumed to be equal in both MZ and DZ twin pairs (Kyvic 2000), whereas the unique environmental component does not contribute to twin similarity, rather it estimates the effects that apply only to each individual including measurement error. Any greater similarity between MZ twins than DZ twins is attributed to greater sharing of genetic influences. Heritability is defined as the proportion of the phenotypic variation attributable to genetic factors, and is given by the equation, *h* ^2^ = (A)/(A + C + E).

## Results

1,003 females from TwinsUK with targeted metabolomic analyses of serum samples using the Biocrates Absolute-IDQ™ kit p150 (163 metabolites) were included in the analysis. Of these, 75 were MZ twin pairs, 228 were DZ twin pairs and 397 were singletons. The mean age of the study sample was 58.5 years (10.45 years SD) and the mean BMI was 25 kg/m^2^ (4.45 kg/m^2^ SD).

We first ran linear regression analysis, adjusting for age and BMI, in the larger population excluding MZ discordant twins.

We studied the correlation between metabolite levels and seven dietary intake patterns: coffee intake, garlic intake and nutritional scores derived from the FFQs summarizing fruit and vegetable intake, alcohol intake, meat intake, hypo-caloric dieting and a “traditional English” diet. These five dietary intake patterns derived from PCA (Teucher et al. 2007) are broadly comparable to those reported in a number of large-scale population-based studies of nutrition, such as the Nurses’ Health cohort in the U.S. (Adebamowo et al. 2005), and the EPIC Postdam study (Schulze et al. 2001). A description of the seven dietary intake patterns and the mean and SD values in the discovery and replication sets is presented in Table 1. We correlated these traits with 163 metabolite levels. 42 metabolite nutrient intake associations were statistically significant with Bonferroni *P* < 0.0003 (Supplementary Table 2), with many associations for both the fruit and vegetable and low meat scores. We then assessed whether these associations with dietary patterns were robust. In order to do so these 42 metabolite nutrient intake associations were tested in the identical twins discordant for the phenotype (i.e. MZ twins with the dietary measure or dietary pattern one SD apart or more). For each dietary variable, we identified between 14 and 20 independent monozygotic pairs discordant for the dietary variable. The regression (beta) coefficients were in the same direction in both analyses (discordant identical twins and the rest of the population) for 11 metabolites. We then combined results using inverse-variance fixed effects meta-analyses. Using a Bonferroni cut-off of 4 × 10^−5^ (0.05/7 nutrients × 163 metabolites), these are shown in Table 2. We find two significant associations with garlic intake, one with coffee intake, six with the dietary pattern for fruit and vegetable, and two with the “dieting” dietary pattern. The remaining 31 associations become not significant after this analysis and we find no validated metabolite associations with three of the five dietary intake patterns derived from FFQs (Table 2). The metabolites associated with nutrient intake and dietary patterns all fall within three broad categories: acylcarnitines, glycerophospholipids and sphingolipids. It is of interest therefore to understand what these metabolites represent and whether they are genetically determined or only environmentally determined. A brief description of pathways to which these metabolites belong are shown in Table 3. In addition, for each of these 11 metabolites, we also calculated the genetic contribution/heritability. The two metabolites negatively correlated with garlic intake are acylcarnitines C8:1 (Octenoylcarnitine, *h* ^2^ = 44 %) and C5–DC(C6–OH)(Glutarylcarnitine, *h* ^2^ = 0 %)—but only one of them shows evidence of a genetic component. Coffee intake was also negatively associated with a metabolite from the acylcarnitines pathway—C10:1(Decenoylcarnitine, *h* ^2^ = 53 %). Finally, we could not compute the ACE model for PC aa C38:6 and PC ae C38:6 as, in both cases, the ICC for DZ was higher than the ICC for MZ, thus violating one of the ACE model assumption and indicating that circulating levels of these metabolites are not genetically determined.

**Table 1:** Food intake and dietary patterns scores in discovery and replication set

Phenotype	Description	Discovery		MZ discordant	
		*N*	Mean(SD)	*N*	Mean(SD)
Coffee intake	Coffee weekly consumption	965	10.24(11.53)	38	15.21(13.28)
Garlic intake	Garlic weekly consumption	975	1.31(2.12)	28	2.73(2.22)
Fruit and vegetable pattern score	Frequent intake of fruit, allium and cruciferous vegetables; low intake of fried potatoes	896	−0.04(1.95)	28	0.88(2.84)
High alcohol pattern score	Frequent intake of beer, wine and allium vegetables; low intake of high fibre breakfast cereals and fruit	884	−0.08(1.38)	40	0.31(1.82)
Traditional English pattern score	Frequent intake of fried fish and potatoes, meats, savoury pies and cruciferous vegetables	896	−0.01(1.34)	28	−0.24(1.17)
Dieting pattern score	Frequent intakes of low-fat dairy products, low-sugar soda; low intake of butter and sweet baked products	896	−0.01(1.25)	28	0.06(1.73)
Low meat pattern score	Frequent intakes of baked beans, pizza and soy food; low intake of meat, other fish and seafood, and poultry	892	−0.12(1.16)	32	−0.12(1.86)

MZ discordant are defined as being one SD apart. This however does not necessarily imply that they are at the population extremes. Hence, mean scores may have opposite signs in the Discovery and MZ discordant cohort. For each food intake and dietary pattern a description of the phenotype is given, along with the number of individuals who entered the analysis in the discovery and MZ discordant cohorts, the mean and standard deviation

**Table 2:** Validated metabolite associations with dietary patterns

Metabolite short name	Nutritional parameter/dietary pattern		Discordant MZ replication				Fixed effect meta-analysis	
		Pathway	*n*	Beta	SE	*P* value	Beta	*P* value
C8:1	Garlic	Acylcarnitines	28	−0.049	0.01	0.001	−0.016	1.80 × 10^−7^
C5–DC(C6–OH)	Garlic	Acylcarnitines	28	−0.017	0.02	0.27	−0.010	1.25 × 10^−5^
C10:1	Coffee	Acylcarnitines	38	−0.001	0.001	0.05	−0.002	3.12 × 10^−6^
C9	Dieting	Acylcarnitines	28	0.007	0.02	0.66	0.026	3.27 × 10^−5^
PC aa C36:6	Fruit and vegetables	Glycerophospholipids	28	0.001	0.01	0.95	0.014	3.78 × 10^−5^
PC aa C38:6	Fruit and vegetables	Glycerophospholipids	28	0.017	0.01	0.03	0.017	1.39 × 10^−9^
PC aa C40:6	Fruit and vegetables	Glycerophospholipids	28	0.023	0.01	0.002	0.014	1.81 × 10^−7^
PC ae C38:6	Fruit and vegetables	Glycerophospholipids	28	0.013	0.01	0.07	0.011	3.92 × 10^−5^
PC ae C40:6	Fruit and vegetables	Glycerophospholipids	28	0.015	0.01	0.05	0.013	1.36 × 10^−6^
PC ae C38:3	Dieting	Glycerophospholipids	28	0.024	0.013	0.09	0.022	3.59 × 10^−5^
SM C26:1	Fruit and vegetables	Sphingolipids	28	0.020	0.01	0.01	0.015	6.95 × 10^−13^

Significant results in MZ discordant twins and meta-analysis (*P* value < 4 × 10^−5^). Beta coefficients, standard errors and *p* value are provided for both the discordant MZ cohort and for the fixed effect meta-analysis

**Table 3:** Heritability of metabolites associated with dietary patterns

Metabolite short name	Metabolite	Pathway	Description	ICC_MZ_	ICC_DZ_	*h* ^2^ (%)	*c* ^2^ (%)	*e* ^2^ (%)
C8:1	Octenoylcarnitine	Acylcarnitines	Facilitate entry of long-chain fatty acids into the mitochondrion and act as fuels for many tissues (e.g. skeletal and cardiac muscle) via fatty acid beta-oxidation	0.51	0.16	44	0	56
C5–DC(C6–OH)	Glutarylcarinitine			0.23	0.20	0	21	79
C10:1	Decenoylcarnitine			0.55	0.26	53	0	47
C9	Nonaylcarnitine			0.60	0.38	62	0	38
PC aa C36:6	Phosphatidylcholine diacyl C36:6	Glycerophospholipids	Composed of glycerol, a phosphate group, and two fatty acid chains, are by far the most abundant lipids in cell membranes	0.43	0.34	54	0	46
PC aa C38:6^a^	Phosphatidylcholine diacyl C38:6			0.21	0.23	–	–	–
PC aa C40:6	Phosphatidylcholine diacyl C40:6			0.49	0.29	57	0	43
PC ae C38:6^a^	Phosphatidylcholine acyl-alkyl C38:6			0.25	0.28	–	–	–
PC ae C40:6	Phosphatidylcholine acyl-alkyl C40:6			0.25	0.20	0	21	79
PC ae C38:3	Phosphatidylcholine acyl-alkyl C38:3			0.40	0.29	38	0	62
SM C26:1	Sphingomyeline C26:1	Sphingolipids	Lipids containing a backbone of sphingoid bases. Important in signal transmission and cell recognition.	0.43	0.15	35	0	65

For each metabolite which was associated overall and in significant MZ twins with (*P* value < 4 × 10^−5^)., we estimated the ICC for MZ and DZ pairs and the additive genetic contribution/heritability (*h* ^2^), the common environmental contribution (*c* ^2^) and the unique environmental contribution (*e* ^2^)

* For this metabolite it was not possible to compute the ACE model as ICC_MZ_ < ICC_D_

With regards to the PCA traits derived from the FFQs, we found the strongest associations with the fruit and vegetables score. Five of the six metabolites significantly and positively correlated with this variable were glycerophospholipids L PC aa C36:6 (Phosphatidylcholine diacyl C36:6, *h* ^2^ = 54 %); PC aa C38:6 (Phosphatidylcholine diacyl C38:6, *h* ^2^ = 0 %); PC aa C40:6 (Phosphatidylcholine diacyl C40:6, *h* ^2^ = 57 %); PC ae C38:6 (Phosphatidylcholine acyl-alkyl C38:6, *h* ^2^ = 0 %); and PC ae C40:6 (Phosphatidylcholine acyl-alkyl C40:6, *h* ^2^ = 57 %). The sixth was a sphingolipid (SM C26:1: Sphingomyeline C26:1, *h* ^2^ = 35 %). Finally, significant positive associations with an acylcarnitine—C9 (Nonacylcarnitine, *h* ^2^ = 62 %) and a glycerophospholipid—PC ae C38:3 (Phosphatidylcholine acyl-alkyl C38:3, *h* ^2^ = 38 %) were found for the hypo-caloric dieting score (Table 3).

## Discussion

In this study we have investigated in a large cross-sectional population of women the role of dietary intake patterns on in metabolomic parameters. Our data support that garlic intake, coffee intake and dietary intake patterns from FFQs representing fruit and vegetables and low calorie intake are correlated with metabolite profiles. All of the associations found fall within three main pathways: acylcarnitines, glycerophospholipids and sphingolipids. However, no association between metabolite levels and meat intake, high alcohol consumption, or the “traditional English” diet were found.

The strongest association identified by our study is between a sphingolipid (Sphingomyeline C26:1 or SM C26:1) and fruit and vegetable intake. Diets rich in fruits and vegetables have been associated with a reduced risk of chronic disease, including cardiovascular disease (Esfahani et al. 2011). Current research indicates that fruit and vegetable concentrates significantly increase serum levels of antioxidant provitamins and vitamins (β-carotene, vitamins C and E) as well as folate, but reduce homocysteine and markers of oxidative stress (Esfahani et al. 2011). Interestingly, sphingolipid depletion has been shown to inhibit vitamin uptake (Stevens and Tang 1997). The other metabolites positively correlated with fruit and vegetable intake are phosphatidylcholines which are highly desaturated fatty acids and among most common constituents of biological membranes (Zhang et al. 2009). They are an essential biological component and widely used as a nutritional supplement for protecting cells from oxidation, increase fat burning and preventing cardiovascular disease (Ristic Medic et al. 2006). Lecithin contains fatty acids identified as the peroxisome proliferator-activated receptor (PPAR) agonists. Experimental data has shown that lecithin promotes adipocyte differentiation and differentiation-specific gene expression and increased triglycerides and free fatty acid levels in the adipocytes (Zhang et al. 2009). Phosphatidylcholine has also been shown to have an anti-inflammatory action (Eros et al. 2009). Therefore, there is a likely physiological link between the metabolites identified by us and the beneficial effects of fruit and vegetable intake which may also be reflected in cardiovascular and other health-related phenotypes.

Our study also confirms the association between coffee consumption with C10:1 which was previously reported by Atlmaier et al. (2009) in a study of German men only and this underlines the reliability and reproducibility of metabolomic data in nutritional research.

A glycerophospholipid is also associated with low-calorie dieting. The positive association between phosphatidylcholine acyl-alkyl C38:3 and “dieting” pattern suggests that increased glycerophospholipid levels may be one possible benefit of a low-calorie diet.

Both garlic and coffee intake were found to be negatively correlated with levels of three acylcarnitines whereas “dieting” was positively correlated with nonacylcarnitine (C9). Acylcarnitines are long-chain fatty acids. Comprehensive plasma acylcarnitine profiles in patients with type 2 diabetes have revealed elevated circulating markers of incomplete long-chain fatty acid catabolism and of acetylcarnitine, together with lower levels of propionylcarnitine. These metabolites appear to be sensitive indicators of biochemical pathways that are responsive to, or underlie, the severity of diabetes and long-term blood sugar control. (Adams et al. 2009). The increased levels of nonacylcarnitine (C9) with low-calorie dieting might thus be reflecting less efficient long fatty acid metabolism. On the other hand, the lower circulating levels of octenoylcarnitine (C8:1) and glutarylcarinitine (C5–DC/C6–0H) with garlic intake and of decenoylcarnitine (C10:1) with associated with coffee intake suggest a possible link between these foods and more efficient mitochondrial fat metabolism. Both garlic (Baños et al. 2008; Padiya et al. 2011) and coffee (Matsuura et al. 2012) have been implicated in decreased risk of metabolic syndrome and improved insulin sensitivity. It is therefore reasonable to hypothesize that the effect of garlic and coffee on acylcarnitines and mitochondrial fat metabolism may be part of the molecular link underlying such clinical observations. The results presented here illustrate the use of metabolomic profiling to gain insights on the role of nutrients on health. But in order to gain a reliable risk estimate with a single blood measurement the within-subject variance over time should be small compared with the between-subject variance. Otherwise the high sensitivity of the metabolome to internal or external stimuli (such as age, hormonal status, diet and lifestyle) may potentially limit their use for risk assessment in large-scale epidemiologic studies that are based on single blood measurement The reliability of the serum metabolite concentrations in the panel used in our study has been previously reported. Floegel et al. (2011) investigated the between- and within-person variation in the metabolite measurements of the 163 serum metabolites in Biocrates panel was analysed the in 100 subjects who had provided two fasting blood samples taken 4 months apart. The metabolite reliability expressed by the ICC (i.e. the ratio of between-person variance and total variance). All the metabolites that we identify as related to dietary intake patterns in our study showed fair to good reliability as defined by Floegel et al. (2011) (values ranging from 0.41 to 0.69) and hence should be reliable for risk assessment in prospective epidemiological studies and useful to researchers investigating the most reliable biomarkers to use in nutritional and healthy lifestyle studies.

Another interesting observation to come out of our study is that two thirds of the metabolites related to nutritional patterns have a significant genetic contribution, but one third have only an environmental contribution. For example, in the case of garlic one of the metabolites has an significant additive genetic component (e.g. octenoylcarnitine C8:1) yet the other metabolite associated appears to be due to environmental influences (e.g. glutarylcarinitine C5–DC/C6–OH).

The present study has several strengths. Firstly, we employed a two-stage analysis design—discovery and independent replication with stringent *P* values—thus minimizing the risk of false positive findings. Secondly, we used identical twins discordant for nutritional intake for the validation analysis. Metabolite levels may be influenced by many factors including genetics (Suhre et al. 2011). Since identical twins share 100 % of their genetic makeup and are matched perfectly for age, gender, social class, etc., we were able to validate the role of nutritional intake on metabolites; isolating the non-genetic contribution. Thus, these data help us understand the complex interplay between genetic and environmental influences that determine nutritional patterns.

The results presented here are consistent with data from the study carried out in men (Altmaier et al. 2011) in that we identify correlations between metabolomic measurements and the intake of certain nutrients and, in particular, nutritional patterns as summarized by principal component analyses. Given the strong results for individual metabolites found by the previous study, we have focused only on individual metabolites and not on metabolite ratios and we confirm that indeed there are very correlations with dietary patterns.

There are some limitations in the current study. Firstly, both our validation and replication samples consist of women only. Some of these metabolites may be influences by sex-hormone levels and results could be different in men. Secondly, we have used FFQs rather than other more reliable methods for assessing nutrient intake.

Over the past two decades, FFQs have become a well-accepted method for quantitative assessment of usual nutrient intake (Sempos et al. 1992) and their for assessing dietary composition has been documented objectively in prospective studies (Willett 1998). FFQ derived dietary intake patterns (using PCA) have been shown repeatedly in the literature to correlate with various health-outcomes such as cardiovascular risk, stroke, diabetes and cancer among others (Stricker et al. 2012; Schulze et al. 2001; Adebamowo et al. 2005; Ouderaa et al. 2006).

Although FFQs may not be perfect, they have been extensively validated for epidemiological studies needing large sample sizes. Notwithstanding, we have been able using FFQs and a targeted metabolomic approach to identify metabolites that are correlated with nutritional patterns and within these, to identify those that have a genetic component from those that are purely environmentally determined.

In conclusion, our results provide further support to the use of metabolomic analyses to identify the molecular mechanisms responsible for the links between nutrition and health. Whereas directly associating lifestyle habits with clinical endpoints provides only limited information about the underlying disease-causing mechanisms, the use of a metabolomic approach helps identify the molecular pathways underlying dietary patterns and these molecular mechanisms in turn can be more easily linked experimentally in smaller studies to relevant clinical outcomes (Jenab et al. 2009; Scalbert et al. 2009; Wishart 2008). Our study confirms that this technology has great potential in the area of nutritional assessment as metabolites reflects certain nutritional patterns and can help separate those metabolites related to nutrient that reflect inherited patterns of metabolism from those that are entirely due to environmental/lifestyle choices. In this context a metabolomic approach may provide useful biomarkers of disease prevention, early disease, or nutritional status, and eventually to identify potential molecular mechanisms in chronic disease processes that are modulated by dietary constituents and/or dietary patterns.

## Electronic supplementary material

Below is the link to the electronic supplementary material.

Supplementary material 1 (DOC 200 kb)

## Abbreviations

BMI: Body mass index
CV: Coefficient of variation
DZ: Dizygotic
EPIC: European prospective investigation into cancer and nutrition
FFQ: Food frequency questionnaire
MZ: Monozygotic
PCA: Principal components analysis
SD: Standard deviation
