# Supplementary material for: Targeted metabolomics profiles are strongly correlated with nutritional patterns in women
Source: Metabolomics. 2012 Oct 6;9(2):506–14. doi: 10.1007/s11306-012-0469-6 (PMC3608890; doi:10.1007/s11306-012-0469-6)
Supplement: Supplementary file 1 — Supplementary material 1 (DOC 200 kb) [file 11306_2012_469_MOESM1_ESM.doc]

**Supplementary Table 1.** List of metabolites

| **Metabolites** | **Short name** | **Pathway** |
| --- | --- | --- |
| Carnitine | C0 | Acylcarnitines |
| Acetylcarnitine | C2 | Acylcarnitines |
| Propionylcarnitine | C3 | Acylcarnitines |
| Propenoylcarnitine | C3:1 | Acylcarnitines |
| Hydroxypropionylcarnitine | C3-OH | Acylcarnitines |
| Butyrylcarnitine | C4 | Acylcarnitines |
| Butenylcarnitine | C4:1 | Acylcarnitines |
| Hydroxypropionylcarnitine | C4-OH(C3-DC) | Acylcarnitines |
| Valerylcarnitine | C5 | Acylcarnitines |
| Tiglylcarnitine | C5:1 | Acylcarnitines |
| Glutaconylcarnitine | C5:1-DC | Acylcarnitines |
| Glutarylcarinitine | C5-DC(C6-OH) | Acylcarnitines |
| Methylglutarylcarnitine | C5-M-DC | Acylcarnitines |
| Hydroxylcarnitine | C5-OH(C3-DC-M) | Acylcarnitines |
| Hexanoylcarnitine | C6(C4:1-DC) | Acylcarnitines |
| Hexenoylcarnitine | C6:1 | Acylcarnitines |
| Pimelylcarnitine | C7-DC | Acylcarnitines |
| Octanoylcarnitine | C8 | Acylcarnitines |
| Octenoylcarnitine | C8:1 | Acylcarnitines |
| Nonaylcarnitine | C9 | Acylcarnitines |
| Decanoylcarnitine | C10 | Acylcarnitines |
| Decenoylcarnitine | C10:1 | Acylcarnitines |
| Decadienylcarnitine | C10:2 | Acylcarnitines |
| Dodecanoylcarnitine | C12 | Acylcarnitines |
| Dodecenoylcarnitine | C12:1 | Acylcarnitines |
| Dodecanedioylcarnitine | C12-DC | Acylcarnitines |
| Tetradecanoylcarnitine | C14 | Acylcarnitines |
| Tetradecenoylcarnitine | C14:1 | Acylcarnitines |
| Hydroxytetradecenoylcarnitine | C14:1-OH | Acylcarnitines |
| Tetradecadienylcarnitine | C14:2 | Acylcarnitines |
| Hydroxytetradecadienylcarnitine | C14:2-OH | Acylcarnitines |
| Hexadecanoylcarnitine | C16 | Acylcarnitines |
| Hexadecenoylcarnitine | C16:1 | Acylcarnitines |
| Hydroxyhexadecenoylcarnitine | C16:1-OH | Acylcarnitines |
| Hexadecadienylcarnitine | C16:2 | Acylcarnitines |
| Hydroxyhexadecadienylcarnitine | C16:2-OH | Acylcarnitines |
| Hydroxyhexadecanoylcarnitine | C16-OH | Acylcarnitines |
| Octadecanoylcarnitine | C18 | Acylcarnitines |
| Octadecenoylcarnitine | C18:1 | Acylcarnitines |
| Hydroxyoctadecenoylcarnitine | C18:1-OH | Acylcarnitines |
| Octadecadienylcarnitine | C18:2 | Acylcarnitines |
| Arginine | Arg-PTC | Amino acids |
| Glutamine | Gln-PTC | Amino acids |
| Glycine | Gly-PTC | Amino acids |
| Histidine | His-PTC | Amino acids |
| Methionine | Met-PTC | Amino acids |
| Ornithine | Orn-PTC | Amino acids |
| Phenylalanine | Phe-PTC | Amino acids |
| Proline | Pro-PTC | Amino acids |
| Serine | Ser-PTC | Amino acids |
| Threonine | Thr-PTC | Amino acids |
| Tryptophan | Trp-PTC | Amino acids |
| Tyrosine | Tyr-PTC | Amino acids |
| Valine | Val-PTC | Amino acids |
| Leucine | xLeu-PTC | Amino acids |
| Hexose | H1 | Sugars |
| lysoPhosphatidylcholine acyl C6:0 | lysoPC a C6:0 | Glycerophospholipids |
| lysoPhosphatidylcholine acyl C14:0 | lysoPC a C14:0 | Glycerophospholipids |
| lysoPhosphatidylcholine acyl C16:0 | lysoPC a C16:0 | Glycerophospholipids |
| lysoPhosphatidylcholine acyl C16:1 | lysoPC a C16:1 | Glycerophospholipids |
| lysoPhosphatidylcholine acyl C17:0 | lysoPC a C17:0 | Glycerophospholipids |
| lysoPhosphatidylcholine acyl C18:0 | lysoPC a C18:0 | Glycerophospholipids |
| lysoPhosphatidylcholine acyl C18:1 | lysoPC a C18:1 | Glycerophospholipids |
| lysoPhosphatidylcholine acyl C18:2 | lysoPC a C18:2 | Glycerophospholipids |
| lysoPhosphatidylcholine acyl C20:3 | lysoPC a C20:3 | Glycerophospholipids |
| lysoPhosphatidylcholine acyl C20:4 | lysoPC a C20:4 | Glycerophospholipids |
| lysoPhosphatidylcholine acyl C24:0 | lysoPC a C24:0 | Glycerophospholipids |
| lysoPhosphatidylcholine acyl C26:0 | lysoPC a C26:0 | Glycerophospholipids |
| lysoPhosphatidylcholine acyl C26:1 | lysoPC a C26:1 | Glycerophospholipids |
| lysoPhosphatidylcholine acyl C28:0 | lysoPC a C28:0 | Glycerophospholipids |
| lysoPhosphatidylcholine acyl C28:1 | lysoPC a C28:1 | Glycerophospholipids |
| Phosphatidylcholine diacyl C24:0 | PC aa C24:0 | Glycerophospholipids |
| Phosphatidylcholine diacyl C26:0 | PC aa C26:0 | Glycerophospholipids |
| Phosphatidylcholine diacyl C28:1 | PC aa C28:1 | Glycerophospholipids |
| Phosphatidylcholine diacyl C30:0 | PC aa C30:0 | Glycerophospholipids |
| Phosphatidylcholine diacyl C30:2 | PC aa C30:2 | Glycerophospholipids |
| Phosphatidylcholine diacyl C32:0 | PC aa C32:0 | Glycerophospholipids |
| Phosphatidylcholine diacyl C32:1 | PC aa C32:1 | Glycerophospholipids |
| Phosphatidylcholine diacyl C32:2 | PC aa C32:2 | Glycerophospholipids |
| Phosphatidylcholine diacyl C32:3 | PC aa C32:3 | Glycerophospholipids |
| Phosphatidylcholine diacyl C34:1 | PC aa C34:1 | Glycerophospholipids |
| Phosphatidylcholine diacyl C34:2 | PC aa C34:2 | Glycerophospholipids |
| Phosphatidylcholine diacyl C34:3 | PC aa C34:3 | Glycerophospholipids |
| Phosphatidylcholine diacyl C34:4 | PC aa C34:4 | Glycerophospholipids |
| Phosphatidylcholine diacyl C36:0 | PC aa C36:0 | Glycerophospholipids |
| Phosphatidylcholine diacyl C36:1 | PC aa C36:1 | Glycerophospholipids |
| Phosphatidylcholine diacyl C36:2 | PC aa C36:2 | Glycerophospholipids |
| Phosphatidylcholine diacyl C36:3 | PC aa C36:3 | Glycerophospholipids |
| Phosphatidylcholine diacyl C36:4 | PC aa C36:4 | Glycerophospholipids |
| Phosphatidylcholine diacyl C36:5 | PC aa C36:5 | Glycerophospholipids |
| Phosphatidylcholine diacyl C36:6 | PC aa C36:6 | Glycerophospholipids |
| Phosphatidylcholine diacyl C38:0 | PC aa C38:0 | Glycerophospholipids |
| Phosphatidylcholine diacyl C38:1 | PC aa C38:1 | Glycerophospholipids |
| Phosphatidylcholine diacyl C38:3 | PC aa C38:3 | Glycerophospholipids |
| Phosphatidylcholine diacyl C38:4 | PC aa C38:4 | Glycerophospholipids |
| Phosphatidylcholine diacyl C38:5 | PC aa C38:5 | Glycerophospholipids |
| Phosphatidylcholine diacyl C38:6 | PC aa C38:6 | Glycerophospholipids |
| Phosphatidylcholine diacyl C40:1 | PC aa C40:1 | Glycerophospholipids |
| Phosphatidylcholine diacyl C40:2 | PC aa C40:2 | Glycerophospholipids |
| Phosphatidylcholine diacyl C40:3 | PC aa C40:3 | Glycerophospholipids |
| Phosphatidylcholine diacyl C40:4 | PC aa C40:4 | Glycerophospholipids |
| Phosphatidylcholine diacyl C40:5 | PC aa C40:5 | Glycerophospholipids |
| Phosphatidylcholine diacyl C40:6 | PC aa C40:6 | Glycerophospholipids |
| Phosphatidylcholine diacyl C42:0 | PC aa C42:0 | Glycerophospholipids |
| Phosphatidylcholine diacyl C42:1 | PC aa C42:1 | Glycerophospholipids |
| Phosphatidylcholine diacyl C42:2 | PC aa C42:2 | Glycerophospholipids |
| Phosphatidylcholine diacyl C42:4 | PC aa C42:4 | Glycerophospholipids |
| Phosphatidylcholine diacyl C42:5 | PC aa C42:5 | Glycerophospholipids |
| Phosphatidylcholine diacyl C42:6 | PC aa C42:6 | Glycerophospholipids |
| Phosphatidylcholine acyl-alkyl C | PC ae C30:0 | Glycerophospholipids |
| Phosphatidylcholine acyl-alkyl C | PC ae C30:1 | Glycerophospholipids |
| Phosphatidylcholine acyl-alkyl C | PC ae C30:2 | Glycerophospholipids |
| Phosphatidylcholine acyl-alkyl C32:1 | PC ae C32:1 | Glycerophospholipids |
| Phosphatidylcholine acyl-alkyl C32:2 | PC ae C32:2 | Glycerophospholipids |
| Phosphatidylcholine acyl-alkyl C34:0 | PC ae C34:0 | Glycerophospholipids |
| Phosphatidylcholine acyl-alkyl C34:1 | PC ae C34:1 | Glycerophospholipids |
| Phosphatidylcholine acyl-alkyl C34:2 | PC ae C34:2 | Glycerophospholipids |
| Phosphatidylcholine acyl-alkyl C34:3 | PC ae C34:3 | Glycerophospholipids |
| Phosphatidylcholine acyl-alkyl C36:0 | PC ae C36:0 | Glycerophospholipids |
| Phosphatidylcholine acyl-alkyl C36:1 | PC ae C36:1 | Glycerophospholipids |
| Phosphatidylcholine acyl-alkyl C36:2 | PC ae C36:2 | Glycerophospholipids |
| Phosphatidylcholine acyl-alkyl C36:3 | PC ae C36:3 | Glycerophospholipids |
| Phosphatidylcholine acyl-alkyl C36:4 | PC ae C36:4 | Glycerophospholipids |
| Phosphatidylcholine acyl-alkyl C36:5 | PC ae C36:5 | Glycerophospholipids |
| Phosphatidylcholine acyl-alkyl C38:0 | PC ae C38:0 | Glycerophospholipids |
| Phosphatidylcholine acyl-alkyl C38:1 | PC ae C38:1 | Glycerophospholipids |
| Phosphatidylcholine acyl-alkyl C38:2 | PC ae C38:2 | Glycerophospholipids |
| Phosphatidylcholine acyl-alkyl C38:3 | PC ae C38:3 | Glycerophospholipids |
| Phosphatidylcholine acyl-alkyl C38:4 | PC ae C38:4 | Glycerophospholipids |
| Phosphatidylcholine acyl-alkyl C38:5 | PC ae C38:5 | Glycerophospholipids |
| Phosphatidylcholine acyl-alkyl C38:6 | PC ae C38:6 | Glycerophospholipids |
| Phosphatidylcholine acyl-alkyl C40:0 | PC ae C40:0 | Glycerophospholipids |
| Phosphatidylcholine acyl-alkyl C40:1 | PC ae C40:1 | Glycerophospholipids |
| Phosphatidylcholine acyl-alkyl C40:2 | PC ae C40:2 | Glycerophospholipids |
| Phosphatidylcholine acyl-alkyl C40:3 | PC ae C40:3 | Glycerophospholipids |
| Phosphatidylcholine acyl-alkyl C40:4 | PC ae C40:4 | Glycerophospholipids |
| Phosphatidylcholine acyl-alkyl C40:5 | PC ae C40:5 | Glycerophospholipids |
| Phosphatidylcholine acyl-alkyl C40:6 | PC ae C40:6 | Glycerophospholipids |
| Phosphatidylcholine acyl-alkyl C42:0 | PC ae C42:0 | Glycerophospholipids |
| Phosphatidylcholine acyl-alkyl C42:1 | PC ae C42:1 | Glycerophospholipids |
| Phosphatidylcholine acyl-alkyl C42:2 | PC ae C42:2 | Glycerophospholipids |
| Phosphatidylcholine acyl-alkyl C42:3 | PC ae C42:3 | Glycerophospholipids |
| Phosphatidylcholine acyl-alkyl C42:4 | PC ae C42:4 | Glycerophospholipids |
| Phosphatidylcholine acyl-alkyl C42:5 | PC ae C42:5 | Glycerophospholipids |
| Phosphatidylcholine acyl-alkyl C44:3 | PC ae C44:3 | Glycerophospholipids |
| Phosphatidylcholine acyl-alkyl C44:4 | PC ae C44:4 | Glycerophospholipids |
| Phosphatidylcholine acyl-alkyl C44:5 | PC ae C44:5 | Glycerophospholipids |
| Phosphatidylcholine acyl-alkyl C44:6 | PC ae C44:6 | Glycerophospholipids |
| Hydroxysphingomyeline C14:1 | SM (OH) C14:1 | Sphingolipids |
| Sphingomyeline C16:0 | SM C16:0 | Sphingolipids |
| Sphingomyeline C16:1 | SM C16:1 | Sphingolipids |
| Hydroxysphingomyeline C16:1 | SM (OH) C16:1 | Sphingolipids |
| Sphingomyeline C18:0 | SM C18:0 | Sphingolipids |
| Sphingomyeline C18:1 | SM C18:1 | Sphingolipids |
| Sphingomyeline C20:2 | SM C20:2 | Sphingolipids |
| Sphingomyeline C22:3 | SM C22:3 | Sphingolipids |
| Hydroxysphingomyeline C22:1 | SM (OH) C22:1 | Sphingolipids |
| Hydroxysphingomyeline C22:2 | SM (OH) C22:2 | Sphingolipids |
| Sphingomyeline C24:0 | SM C24:0 | Sphingolipids |
| Sphingomyeline C24:1 | SM C24:1 | Sphingolipids |
| Hydroxysphingomyeline C24:1 | SM (OH) C24:1 | Sphingolipids |
| Sphingomyeline C26:0 | SM C26:0 | Sphingolipids |
| Sphingomyeline C26:1 | SM C26:1 | Sphingolipids |

**Supplementary Table 2. Significant metabolite nutrient intake associations adjusted for age and BMI*. Beta coefficients, standard errors and p-value are provided.**

| **Metabolite** | **Dietary intake pattern** | **Beta** | **SE** | **p-value** |
| --- | --- | --- | --- | --- |
| C8:1 | Garlic | -0.01387 | 0.003201 | 1.49x10^-5^ |
| C5-DC(C6-OH) | Garlic | -0.00992 | 0.00234 | 2.24x10^-5^ |
| C16-OH | Garlic | -0.00931 | 0.002453 | 1.45x10^-4^ |
| C10:1 | Coffee | -0.00182 | 0.000421 | 1.56x10^-5^ |
| PC aa C36:5 | Fruit and Vegetables | 0.014409 | 0.003933 | 2.52x10^-4^ |
| PC aa c36:6 | Fruit and Vegetables | 0.016172 | 0.003665 | 1.03x10^-5^ |
| PC aa C38:6 | Fruit and Vegetables | 0.016566 | 0.002989 | 3.02x10^-8^ |
| PC aa C40:6 | Fruit and Vegetables | 0.013215 | 0.002853 | 3.66x10^-6^ |
| PC ae C38:6 | Fruit and Vegetables | 0.0103 | 0.00283 | 2.73x10^-4^ |
| PC ae C40:0 | Fruit and Vegetables | 0.00865 | 0.00238 | 2.83x10^-4^ |
| PC ae C40:6 | Fruit and Vegetables | 0.012057 | 0.002766 | 1.30x10^-5^ |
| SM C26:1 | Fruit and Vegetables | 0.014426 | 0.002992 | 1.44x10^-6^ |
| C6:1 | High Alcohol | -0.00057 | 0.000156 | 2.73x10^-4^ |
| PC aa C42:0 | High Alcohol | -0.01512 | 0.004096 | 2.07x10^-4^ |
| SM C20:2 | High Alcohol | -0.04252 | 0.009543 | 8.20x10^-6^ |
| C16-OH | Dieting | 0.020848 | 0.0048006 | 1.42x10^-5^ |
| C9 | Dieting | 0.0257621 | 0.0060173 | 1.87x10^-5^ |
| PC ae C36:2 | Dieting | 0.0167202 | 0.0046723 | 3.44x10^-4^ |
| PC ae C38:3 | Dieting | 0.0214528 | 0.0058104 | 2.24x10^-4^ |
| C0 | Low Meat | -0.01496 | 0.00354 | 2.70x10^-5^ |
| C5 | Low Meat | -0.01915 | 0.004852 | 8.80x10^-5^ |
| C5:1 | Low Meat | -0.01841 | 0.004835 | 1.50x10^-4^ |
| PC aa C28:1 | Low Meat | -0.01645 | 0.004366 | 1.80x10^-4^ |
| PC aa C30:0 | Low Meat | -0.01832 | 0.005026 | 2.90x10^-4^ |
| PC aa C36:0 | Low Meat | -0.03115 | 0.005325 | 7.90x10^-9^ |
| PC aa C36:5 | Low Meat | -0.05159 | 0.00657 | 1.70x10^-14^ |
| PC aa C36:6 | Low Meat | -0.04258 | 0.005976 | 2.80x10^-12^ |
| PC aa c38:0 | Low Meat | -0.02194 | 0.005019 | 1.40x10^-5^ |
| PC aa C38:5 | Low Meat | -0.02037 | 0.004342 | 3.30x10^-6^ |
| PC aa C38:6 | Low Meat | -0.0315 | 0.005238 | 3.00x10^-9^ |
| PC aa C40:6 | Low Meat | -0.02942 | 0.005251 | 3.1010^-8^ |
| PC aa C42:6 | Low Meat | -0.0147 | 0.004078 | 3.40x10^-4^ |
| PC ae C32:2 | Low Meat | -0.01865 | 0.004151 | 8.30x10^-6^ |
| PC ae C34:0 | Low Meat | -0.02284 | 0.005073 | 8.00x10^-6^ |
| PC ae C36:5 | Low Meat | -0.02312 | 0.004583 | 5.90x10^-7^ |
| PC ae C38:0 | Low Meat | -0.03166 | 0.006433 | 1.10x10^-6^ |
| PC ae C38:6 | Low Meat | -0.02815 | 0.004476 | 5.90x10^-10^ |
| PC ae C40:2 | Low Meat | -0.02092 | 0.004907 | 2.30x10^-5^ |
| PC ae C40:6 | Low Meat | -0.02358 | 0.004359 | 8.90x10^-8^ |
| SM (OH) C16:1 | Low Meat | -0.01628 | 0.003974 | 4.70x10^-5^ |
| SM C18:0 | Low Meat | -0.01307 | 0.00361 | 3.20x10^-4^ |
| SM C26:1 | Low Meat | -0.02478 | 0.004916 | 6.00x10^-7^ |
